# Identification of the complete coding cDNAs and expression analysis of *B4GALT1, LALBA, ST3GAL5, ST6GAL1* in the colostrum and milk of the Garganica and Maltese goat breeds to reveal possible implications for oligosaccharide biosynthesis

**DOI:** 10.1186/s12917-019-2206-0

**Published:** 2019-12-18

**Authors:** Alessandra Crisà, Salvatore Claps, Bianca Moioli, Cinzia Marchitelli

**Affiliations:** 1 CREA - Research Centre for Animal Production and Acquaculture, Via Salaria, 31, 00015 Monterotondo, RM Italy; 2 S.S. 7 Via Appia, 85051 Bella Muro, PZ Italy

**Keywords:** Oligosaccharides, RT-qPCR, *B4GALT1*, *ST3GAL5*, *ST6GAL1*, *LALBA*, Milk somatic goat cells, Colostrum cells

## Abstract

**Background:**

Milk sialylated oligosaccharides (SOS) play crucial roles in many biological processes. The most abundant free SOS in goat’s milk are 3’sialyllactose (3′-SL), 6’sialyllactose (6′-SL) and disialyllactose (DSL). The production of these molecules is determined genetically by the expression of glycosyltransferases and by the availability of nucleotide sugar substrates, but the precise mechanisms regulating the differential patterns of milk oligosaccharides are not known. We aimed to identify the complete cDNAs of candidate genes implicated in SOS biosynthesis (*B4GALT1, LALBA, ST3GAL5, ST6GAL1*) and to analyse their expression during lactation in the Garganica and Maltese goat breeds. Moreover, we analysed the colostrum and milk contents of 3′-SL, 6′-SL and disialyllactose (DSL) and the possible correlations between expressed genes and SOS.

**Results:**

We identified the complete coding cDNAs of *B4GALT1* (HQ700335.1), *ST3GAL5* (KF055858.2), and *ST6GAL1* (HQ709167.1), the single nucleotide polymorphism (SNPs) of these genes and 2 splicing variants of the *ST6GAL1* cDNA. RT-qPCR analysis showed that *LALBA* and *ST6GAL1* were the genes with the highest and lowest expression in both breeds, respectively. The interaction effects of the breeds and sampling times were associated with higher levels of *B4GALT1* and *ST3GAL5* gene expression in Garganica than in Maltese goats at kidding. *B4GALT1, LALBA,* and *ST3GAL5* gene expression changed from kidding to 60 and 120 days in Maltese goats, while in Garganica goats, a difference was observed only for the *LALBA* gene. Breed and lactation effects were also found for SOS contents. Positive correlations of *B4GALT1*, *LALBA, ST3GAL5*, and *ST6GAL1* with 3′-SL/6′SL and DSL were found.

**Conclusions:**

The genetic effect on the oligosaccharide content of milk was previously highlighted in bovines, and this study is the first to investigate this effect in two goat breeds (Garganica and Maltese) during lactation. The genetic variability of candidate genes involved in SOS biosynthesis highlights their potential role in affecting gene expression and ultimately biological function. The investigation of gene regulatory regions as well as the examination of other sialyltransferase genes will be needed to identify the genetic pattern leading to a higher SOS content in the autochtonous Garganica breed and to protect it using a focused breeding strategy.

## Background

Carbohydrates and, in particular, their oligosaccharidic fraction are part of the plethora of milk components that have functional health benefits for consumers. Oligosaccharides (OS) are synthesized in the mammary gland, contain between 3 and 10 monosaccharides, and can be divided into neutral and acidic fractions. The five principal monosaccharides component of milk OS are: D-glucose (Glc), D-galactose (Gal), N-acetylglucosamine (GlcNAc), L-fucose (Fuc), and sialic acid (Sia, specifically N-acetylneuraminic acid [Neu5Ac] in humans and both Neu5Ac and N-glycolyl neuraminic acid [Neu5Gc] in most other species). Lactose (Galb1-4Glc) forms the reducing end of milk OS; 3′-SL and 6′-SL can be synthesized by the addiction of Sia to Gal in α2–3 and/or α2–6 linkages, respectively. Lactose can also be fucosylated in α1–2, α1–3 linkages to form 2’fucosyllactose (2-FL) and 3’fucosyllactose (3′-FL), respectively. These trisaccharides are referred to as short-chain milk OS [1, 2]. Moreover, if a sialic acid is added in an α-2-8 linkage to 3′-SL, the disialyllactose (DSL) tetrasaccharides is formed.

It is generally accepted that most OS are resistant to the pH of the stomach in infants; they are also resistant to enzymatic hydrolysis in the small intestine and are thus largely undigested and unabsorbed. Therefore, most OS will pass through the intestinal tract and enter the colon intact [3].

Different healthful properties have been attributed to OS; they exhibit several protective and physiological roles, including showing prebiotic activity and inhibition of pathogen adhesion in the gastrointestinal tract of infants, immunoregulation, modulation of the microbiota in neonates, intestinal barrier protection, and participation in cognitive function development. The biological functions of OS in human health and nutrition have been demonstrated by a vast number of functional in vitro and in vivo studies [4–7].

OS concentrations is high in the colostrum (up to 50 g/L or more) with an average of 10–15 g/L in mature human milk [8]. Compared with humans, the concentration of OS in the milk of the most relevant domestic species is lower by a factor of 10 to 100 [9]. Moreover, higher OS concentrations have been reported in the colostrum than during late lactation in different animal species [10–12].

Goats produce milk containing free milk OS at much higher levels than cattle or sheep and with a variability, similar to those of human milk [13, 14]. Therefore goat milk also present potential as a supplement for infant formulae and for the development of functional foods or animal feedstuffs on an industrial scale [15, 6]. The recovery and exploitation of whey (rich in OS) by membrane technology during cheesemaking may add value and encourage a decrease in environmental pollution [14–19].

In the last decade, scientists moved beyond the investigation of the components of milk itself and examined the genomics, transcriptomics and proteomics of milk; an example is provided by the Milk Genomics Consortium [20]. To understand the complex biology of milk OS metabolism, it is important to identify the genes that encode glycosylation-related enzymes and, in human, some investigation on glycosylation-related genes and their expression have been carried out [21, 22]. Harduin-leper [23] reported a comprehensive analysis of sialyltransferase in vertebrate genomes; however, the mechanisms regulating differential gene expression in the mammary gland or milk somatic cells of dairy animals are still almost unknown. Sialyltransferases are a subset of glycosyltransferases that use cytidine-5′-monophospho-N-acetylneuraminic acid (CMP-Neu5Ac) as an activated sugar donor to catalyse the transfer of sialic acid residues to terminal non-reducing positions in the OS chains of glycoproteins and glycolipids. They catalyse the formation of different linkages (α2–3, α2–6, and α2–8) and differ in their acceptors. They have been grouped into four families according to the carbohydrate linkages that they synthesize: α 2–3-syaliltransferase (ST3Gal I-VI), α 2–6-syaliltransferase (ST6GALI-II), GalNAc α 2–6-syaliltransferase (ST6GalNAc I-VI), α 2–8- syaliltransferase (ST8Sia I-VI) [24–26].

Beyond physiological (age, kidding, body weight, stage, and number of lactations), environmental and management factors, milk characteristics are influenced by animal genetics (breed and genotype) [27, 28].

The study of OS biosynthesis genes and the identification of polymorphisms associated with naturally occurring OS types, in economically important dairy species and breeds, represents the first step to plan genetic improvement programs and to forecast technological/industrial applications.

In both cattle [12, 29–31] and goats [28, 32], differences in the OS profiles of milk have been found between breeds, and a higher concentration of sialylated oligosaccharides (SOS) fractions has been reported.

Dairy goats are of economic and social importance in southern Italy due to their ability to use vegetation in marginal areas. The Maltese goat represents the lactating goat “*par excellence*” of the Mediterranean, and the trinomial “rusticity- absence of horns- milk production” is still valid. The absence of horns plays a fundamental role in intensive and semi-intensive breeding. The Garganica breed is an autochthonous breed of economic importance on the Gargano Promontory. It represents a wager in the optics of sustainable and multifunctional breeding (environmental protection), particularly in internal and mountain areas, and the characterization of its milk and derivatives is a key element for the protection of the breed.

We focused on the free OS fraction of goat milk and, in particular, the sialylated fraction; these prebiotic molecules have been shown to have many beneficial effects in the human body and, overall, in the gut [33, 34]. A first crucial step is to determine which glycosylation-related genes are involved in the biosynthesis of OS in goat milk somatic cells.

The aims of this study are as follows: 1) identification of the complete cDNAs of three candidate genes potentially implicated in SOS biosynthesis. We decided to study beta-1,4-galactosyltransferase 1 (*B4GALT1)* and lactalbumin alpha *(LALBA)* related to lactose synthesis (the precursor molecule for 3′-SL and 6′-SL biosynthesis), beta-galactoside alpha-2,3-sialyltransferase 5 (*ST3GAL5*) related to 3′-SL biosynthesis and beta-galactoside alpha-2,6-sialyltransferase 1 (*ST6GAL1*) related to 6′-SL biosynthesis; 2) expression analysis of *B4GALT1, LALBA*, *ST3GAL5,* and *ST6GAL1* by RT-qPCR in the goat colostrum/milk somatic cells (GMSCs) of Garganica and Maltese breeds at four lactation stages (kidding, 1, 60, 120 days); and 3) investigation of 3′-SL, 6′-SL and DSL contents and correlation analysis of the different studied phenotypes (gene expression and/or SOS).

## Results

### Sequencing analysis of the *B4GALT1*, *ST3GAL5* and *ST6GAL1* genes

In this work, we identified the expressed complete coding cDNAs of three genes potentially involved in oligosaccharide metabolism. For DSL, we designed primers for the α-N-acetyl-neuraminide α-2,8-sialyltransferase 6 (*ST8SIA6*) gene [35], but we encountered problems with cDNA amplification, and we did not study this gene further (data not shown).

The *B4GALT1* coding sequence obtained up from the assembly of two PCR amplicons; it is 1508 bp long, gives rise to an open reading of 1209 nucleotides, is composed of six exons and encodes a polypeptide of 402 amino acids. Two initial ATG codons are present, similar to the known mammalian *B4GALT1* long isoform of the transcript. The sequence was deposited in GenBank under accession number HQ700335.1. The cDNA sequences from 8 Maltese milk samples revealed the presence of 2 SNPs in the 3’UTR (n.1294 G > A; n.1348 G > A). The deduced protein sequences showed identity with the four functional domains of the B4GalT-1 protein; comparison with the bovine sequence showed an overall similarity of 95.52%, with 18 amino acid differences throughout the coding sequences.

The complete *ST3GAL5* cDNA was compiled by merging the sequences of three amplicons; it is 1358 bp long, gives rise to an open reading frame of 1263 nucleotides, is composed of seven exons and encodes a protein of 420 amino acids. Comparison of the predicted ST3GAL5 goat protein with the bovine protein showed 97% identity and 13 amino acid differences throughout the coding sequences. The sequence was deposited in GenBank under accession number KF055858.2. Sequencing of the cDNAs from 8 Maltese milk samples showed the presence of 2 SNPs: c.213 G > A in exon 2 and c.240 T > C in exon 3. The first causes an amino acid change from Gly to Asp (aa67), and the second causes an amino acid change from Phe to Ser (aa76). The SIFT analysis predicted that Gly67Asp in exon 2 is tolerated (score 0.329), while Phe76Ser substitution in exon 3 is predicted to be deleterious for protein function (score 0.00).

**Fig. 1:**
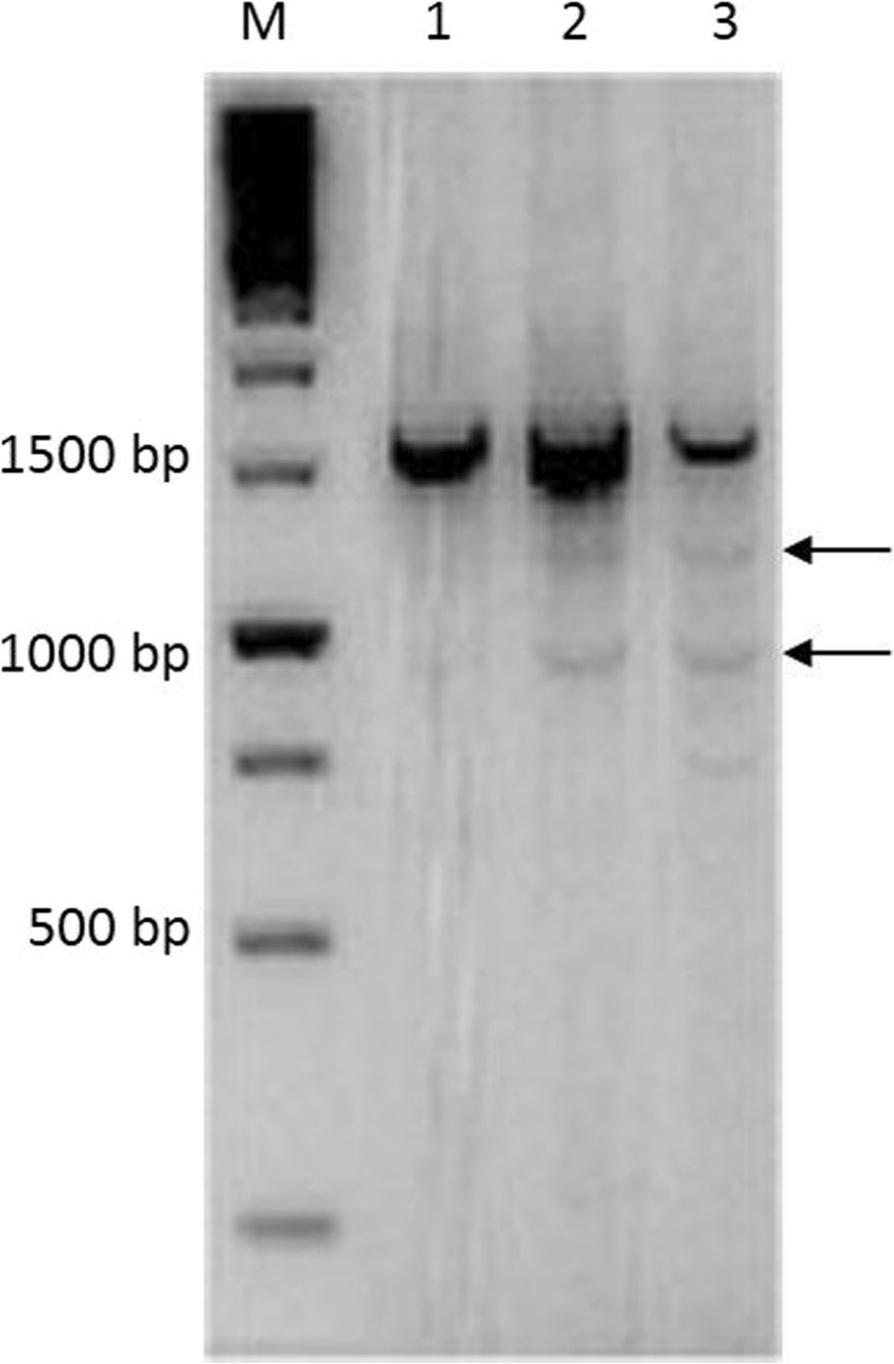
Agarose gel electrophoresis (1,5%) of *ST6GAL1* cDNA (1593 bp amplicon) from the milk somatic cells of three goats. Different lanes correspond to amplicons obtained from the milk somatic cells of three goats. M = GeneRuler1 kb DNA ladder (Thermofisher Scientific). Arrows indicate the sequenced splicing fragments

The complete *ST6GAL1* cDNA was compiled from the assembly of two PCR amplicons; it is 1672 bp long, spans a coding region of 1218 nucleotides, is composed of five coding exons and encodes a protein of 414 amino acids. Comparison of the predicted ST6GAL1 goat protein with the bovine protein showed 98% similarity and 10 amino acid differences, most of which were located in the first 90 amino acids. The sequence was deposited in GenBank under accession number HQ709167.1. In Fig. 1, the results of PCR gel electrophoresis of the 1593 bp amplicon from the cDNAs of 3 Maltese milk samples are shown. Sequences analysis of the weak bands indicated by arrows showed that the slower band corresponds to a cDNA with a spliced coding exon 2 and the faster band to a cDNA with a spliced coding exon 1.

Sequencing of cDNA from 8 Maltese milk samples showed the presence of 4 SNPs: c.372 T > A and c.533 G > A in exon 1, c.977 C > T in exon 4 and c.1310 C > T in exon 5. The first three SNPs cause synonymous amino acid changes, while the c.372 T > A SNP causes a Ser versus Thr amino acid change (aa69). The SIFT analysis predicted this substitution in exon 1 to be tolerated for protein function (score 0,59).

### Gene expression analysis of *B4GALT1*, *LALBA*, *ST3GAL5* and *ST6GAL1* during lactation

In this work, we aimed to study the expression of four genes *(B4GALT1, LALBA, ST3GAL5, ST6GAL1)* during lactation in two genetically distant goat breeds characterized by different productive traits. As an initial step, optimization of the primer concentration, annealing temperature and PCR efficiency was performed for all primer pairs used for both target and RGs. The results showed that a PCR efficiency of 92–105% of was achieved with the settled parameters (Additional file 1). The geNormPLUS analysis showed that *ATP5B* and *SDH* were the two most stable RGs (Additional file 2). For one MA and two GA animals, the 120-day sampling time point could not be analysed (dry-off). All procedures followed for the qPCR experiments complied with the Minimum Information for the Publication of Quantitative Real-time PCR Experiments (MIQE) guidelines [36].

**Fig. 2:**
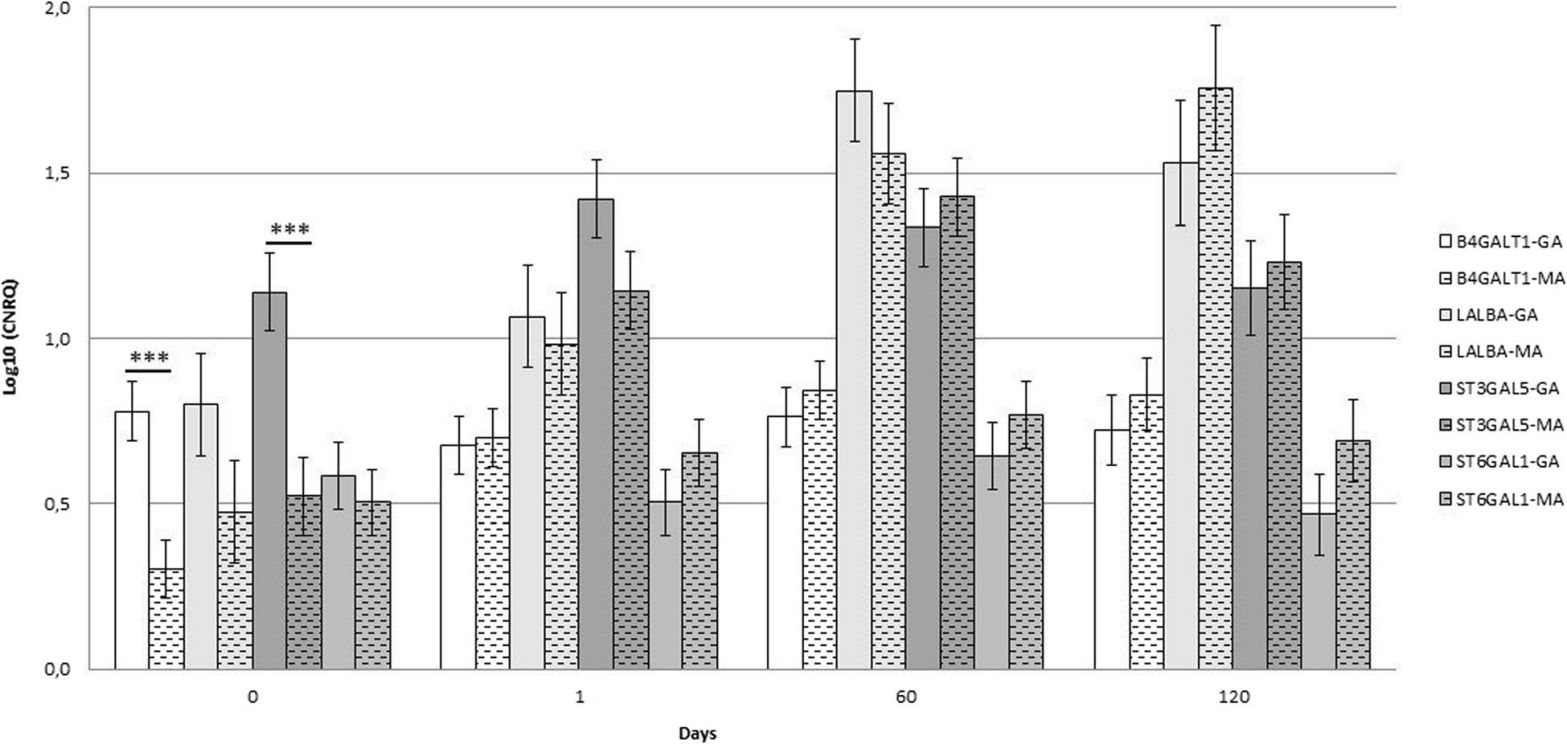
*B4GALT1, LALBA, ST3GAL5,* and *ST6GAL1,* gene expression in the Garganica (GA) and Maltese (MA) breeds during lactation. Lsmean values of log10-transformed cumulative normalized relative quantities (CNRQ) for the *B4GALT1, LALBA, ST3GAL5* and *ST6GAL1* genes in Garganica (GA) and Maltese (MA) somatic cell samples in colostrum (0,1 days) and milk (60,120 days). Uniform and dashed coloured boxes represent the Garganica and Maltese breeds, respectively. Statistically significant differences in the expressed genes between breeds are represented. *** P < 0.001

The effects of breed and lactation on gene expression are reported in Tables 1, while the effects of the interaction between the breeds and sampling times on gene expression are reported in Table 2 and Fig. 2 (please insert Table 2 after line 197). In Fig. 2, statistically significant differences between breeds are reported.

**Table 1:** Effect of breed and sampling time on log10-transformed *B4GALT1, LALBA, ST3GAL5,* and *ST6GAL1* CNRQ values

	Breed		Sampling time (days)			
	GA	MA	0	1	60	120
Gene	Lsmean ± SE	Lsmean ± SE	Lsmean ± SE	Lsmean ± SE	Lsmean ± SE	Lsmean ± SE
*B4GALT1*	0.736 ± 0.04	0.668 ± 0.04	0.542 ± 0.06^b^	0.688 ± 0.06^ab^	0.802 ± 0.06^a^	0.776 ± 0.07^ab^
*LALBA*	1.286 ± 0.08	1.193 ± 0.08	0.637 ± 0.10^B^	1.024 ± 0.10^B^	1.653 ± 0.10^A^	1.644 ± 0.13^A^
*ST3GAL5*	1.262^a^ ± 0,06	1.081^b^ ± 0,06	0.831 ± 0,08^B^	1.281 ± 0.08^A^	1.381 ± 0.08^A^	1.191 ± 0.10^A^
*ST6GAL1*	0,551 ± 0.05	0,654 ± 0.05	0,545 ± 0,07	0.579 ± 0.07	0.706 ± 0.07	0.580 ± 0.08

Lsmean = estimated mean

SE = standard error

Uppercase superscripts within rows indicate significantly different LS means at *P* < 0.001

Lowercase superscript within columns indicates significantly different LS means at *P* < 0.01

CNRQ = calibrated normalized relative quantities

*B4GALT1* = β-1,4-galactosyltransferase 1

*LALBA* = lactalbumin-α

*ST3GAL5* = β-galactoside α-2,3-sialyltransferase 5

*ST6GAL1* = β-galactoside α-2,6-sialyltransferase 1

**Table 2:** Effect of interaction between breed and sampling time on log10 transformed *B4GALT1, LALBA, ST3GAL5, ST6GAL1* CNRQ values

		Sampling time (days)													
		0		1		60		120							
Gene	Breed	Lsmean ± SE	P	Lsmean ± SE	P	Lsmean ± SE	P	Lsmean ± SE	P	P0 > 1	P0 > 60	P0 > 120	P1 > 60	P1 > 120	P60 > 120
*B4GALT1*	GA	0.78 ± 0.09^a^	0.01	0.68 ± 0.09	n.s.	0.76 ± 0.09	n.s.	0.72 ± 0.10	n.s.	n.s.	n.s	n.s	n.s.	n.s.	n.s.
*B4GALT1*	MA	0.30 ± 0.09^Bb^		0.70 ± 0.09^AB^		0.84 ± 0.09 ^A^		0.83 ± 0.10 ^A^		n.s.	0.0026	0.00116	n.s.	n.s.	n.s.
*LALBA*	GA	0.80 ± 0.15^B^	n.s.	1.07 ± 0.15 ^AB^	n.s.	1.75 ± 0.15 ^A^	n.s.	1.53 ± 0.18 ^AB^	n.s.	n.s.	0.0025	n.s	n.s.	n.s.	n.s.
*LALBA*	MA	0.47 ± 0.15^B^		0.98 ± 0.15 ^AB^		1.56 ± 0.15 ^A^		1.75 ± 0.18 ^AC^		n.s.	0.0005	0.00028	n.s.	n.s.	n.s.
*ST3GAL5*	GA	1.14 ± 0.11^a^	0.014	1.42 ± 0.11	n.s.	1.33 ± 0.11	n.s.	1.15 ± 0.14	n.s.	n.s.	n.s.	n.s.	n.s.	n.s.	n.s.
*ST3GAL5*	MA	0.52 ± 0.11^Bb^		1.14 ± 0.11^A^		1.42 ± 0.11^A^		1.23 ± 0.14 ^A^		0.013	0.0002	0.01	n.s.	n.s.	n.s.
*ST6GAL1*	GA	0.58 ± 0.10	n.s.	0.50 ± 0.10	n.s.	0.64 ± 0.10	n.s	0.46 ± 0.12	n.s	n.s.	n.s.	n.s	n.s.	n.s.	n.s.
*ST6GAL1*	MA	0.50 ± 0.10		0.65 ± 0.10		0.77 ± 0.10		0.69 ± 0.12		n.s.	n.s.	n.s	n.s.	n.s.	n.s.

Lsmean = estimated mean

SE = standard error

n.s. = not significant

Uppercase superscripts within rows indicates significantly different LS means between sampling times

Lowercase superscript within columns indicates significantly different LS means between breeds

*P*-values of the differences between sampling times are reported in the last six columns

CNRQ = Calibrated normalized relative quantities

*B4GALT1* = β-1,4-galactosyltransferase 1

*LALBA* = lactalbumin-α

*ST3GAL5* = β-galactoside α-2,3-sialyltransferase 5

*ST6GAL1* = β-galactoside α-2,6-sialyltransferase 1

*LALBA* was the most highly expressed gene, followed by *ST3GAL5, B4GALT1* and *ST6* (1.206, 1.171, 0.695, and 0.6 mean values, respectively). A breed effect was found for *ST3GAL5,* whose expression was higher in GA. Lactation effects were shown for all genes except for *ST6GAL1.* In particular, *B4GALT1* expression increased from 0 to 60 days, *LALBA* expression increased from 0 and 1 day to 60 and 120 days, and *ST3GAL5* expression increased from 0 to 1, 60 and 120 days (please insert here Table 2.

The breed/sampling time interaction effects of *B4GALT1* and *ST3GAL5* at day 0 were stronger in the GA breed (*p* = 0.01 and *p* = 0.014, respectively). During lactation, *B4GALT1* gene expression increased from 0 to 60 and 120 days only in the MA breed (*p* = 0.0026 and 0.0016, respectively). *LALBA* showed an increase from 0 to 60 days in the GA breed (*p* = 0.0025) and from 0 to 60 and 120 days in the MA breed (p = 0.0025 and 0.00028, respectively). *ST3GALT5* expression increased from day 0 to 1, 60 and 120 days in the MA breed (0.0002 ≤ *p* ≤ 0.01). No difference was observed for the *ST6GAL1* gene between either lactation stages or breeds.

### Evaluation of oligosaccharide contents in colostrum and milk

Overall, 3′-SL was the most abundant SOS, followed by DSL and 6′-SL (170.77 and 52 mg/L mean values, respectively). The evaluation of SOS contents in colostrum and milk showed a significant effect of the breed and sampling time (Table 3).

**Table 3:** Effects of breed and sampling time on SOS content

	Breed		Sampling time (days)			
	GA	MA	0	1	60	120
SOS	Lsmean ± SE	Lsmean ± SE	Lsmean ± SE	Lsmean ± SE	Lsmean ± SE	Lsmean ± SE
3′-SL	167.48 ± 14.34	146.02 ± 14.34	253.09 ± 19.13^A^	303.12 ± 19.13^A^	59.822 ± 19.13^B^	10.989 ± 23.42^B^
6′-SL	52.95 ± 5.01	43.19 ± 5.01	55.05 ± 6.69^B^	115.45 ± 6.69^A^	15.256 ± 6.69^BC^	6.558 ± 8.19^BC^
DSL	52.39^B^ ± 5.11	93.24^A^ ± 5.11	129.69 ± 6.82^A^	112.72 ± 6.82^A^	23.043 ± 6.82^B^	25.827 ± 8.35^B^

The SOS concentration is reported as mg/L

Lsmean = estimated mean

SE = standard error

Uppercase superscripts within rows indicate significantly different LS means at *P* < 0.001

SOS = sialyloligosaccharides

6′-SL = 6’sialyllactose

3′SL = 3’sialyllactose

DSL = disialyllactose

A breed effect was shown for DSL at a higher concentration in MA. A sampling time effect was observed for all the SOS. In particular, the 3′-SL content decreased from 0 and 1 days to 60 and 120 days; the 6′-SL content increased from 0 to 1 days and then decreased from 60 and 120 days; and the DSL content decreased from 0 and 1 days to 60 and 120 days.

Considering the interaction effect (Table 4), differences between breeds were observed at 1 day, when 3′-SL values were higher in GA (*p* = 0.032), and DSL was higher in MA (*p* = 0.0001). (please insert here Table 4). During lactation, decreases in the contents of all SOS were observed from 1 to 60 days in both breeds, whereas there was an increase in 6′-SL from 0 to 1 days. The 3′-SL content increased from 0 to 1 day only in GA, while DSL decreased.

**Table 4:** Effect of interaction between breed x sampling time on SOS content

	Sampling time (days)													
	0		1		60		120							
SOS	Lsmean ± SE	P	Lsmean ± SE	P	Lsmean ± SE	P	Lsmean ± SE	P	P0 > 1	P0 > 60	P0 > 120	P1 > 60	P1 > 120	P60 > 120
3′-SL-GA	244.81 ± 27.05^B^	n.s.	367.97 ± 27.05^Aa^	0.032	46.60 ± 27.05^C^	n.s	10.56 ± 33.13^CD^	n.s	0.049	0.0003	0.0002	< 0.0001	< 0.0001	n.s
3′-SL-MA	261.37 ± 27.05^A^		238.26 ± 27.05^Ab^		73.04 ± 27.05^B^		11.41 ± 33.13^B^		n.s	0.0005	< 0.0001	0.0027	0.0026	n.s.
6′-SL-GA	60.11 ± 9.46^B^	n.s.	135.79 ± 9.46^A^	n.s.	3.86 ± 9.46^C^	n.s.	12.07 ± 11.58^BC^	n.s.	0.00017	0.0038	n.s.	< 0.0001	< 0.0001	n.s
6′-SL-MA	49.98 ± 9.46^B^		95.11 ± 9.46^A^		26.65 ± 9.46^BC^		1.05 ± 11.58^C^		0.034	n.s.	0.043	0.00037	< 0.0001	n.s
DSL-GA	132.19 ± 9.64^A^	n.s.	54.44 ± 9.64^Bb^	0.0001	4.92 ± 9.64^C^	n.s.	18.09 ± 11.81^BC^	n.s.	0.00019	< 0.0001	< 0.0001	0.017	n.s.	n.s
DSL-MA	127.26 ± 9.64^A^		170.99 ± 9.64^Aa^		41.16 ± 9.64^B^		33.57 ± 11.81^B^		n.s.	< 0.0001	< 0.0001	< 0.0001	< 0.0001	n.s.

SOS = sialyloligosaccharides

The SOS concentration is reported as mg/L

Uppercase superscripts within rows indicate significantly different LS means between sampling times

Lowercase superscripts within columns indicate significantly different LS means between breeds

P-values of the differences between sampling times are reported in the last six columns

Lsmean = estimated mean

SE = standard error

6′-SL = 6’sialyllactose

3′SL = 3’sialyllactose

DSL = disialyllactose

### Correlation analysis between the analysed phenotypes

Pearson’s correlation coefficients between the studied phenotypes throughout the experiment (gene expression levels and SOS contents) are shown in Table 5.

**Table 5:** Pearson’s correlation coefficients between the levels of gene expression and sialyl oligosaccharides throughout the experiment

	*LALBA*	*ST3GAL5*	*ST6GAL1*	6′-SL	3′-SL	DSL
*B4GALT1*	0.46139**	0.35082*	0.51407***	− 0.07286	− 0.37569*	− 0.26596
*LALBA*		0.32301*	0.11308	−0.43280**	− 0.54178***	− 0.64733***
*ST3GAL5*			0.21658	0.06751	− 0.13598	− 0.34050*
*ST6GAL1*				−0.22778	− 0.24191	0.04724
6′-SL					0.72745***	0.42474**
3′-SL						0.55351***
DSL						

Pearson coefficient significance: * *P* < 0.05; ** *P* < 0.01; *** *P* < 0.001

*B4GALT1* = β-1,4-galactosyltransferase 1

*LALBA* = lactalbumin-α

*ST3GAL5* = β-galactoside α-2,3-sialyltransferase 5

*ST6GAL1* = β-galactoside α-2,6-sialyltransferase 1

6′-SL = 6’sialyllactose

3′SL = 3’sialyllactose

DSL = disialyllactose

Regarding gene expression, we observed moderate statistically significant correlations between *B4GALT1* and *LALBA* (r = 0.46139; *p* = 0.0014) and between *B4GALT1* and *ST6GAL1* (r = 0.51407; *p* = 0.0003). Regarding SOS, a strong correlation between 6′-SL and 3′-SL (r = 0.72745; *p* < 0.0001) and moderate correlations between 6′-SL and DSL (r = 0.42474; *p* = 0.0041) and between 3′-SL and DSL (r = 0.55351; p < 0.0001) were found. Finally, the correlations between gene expression and oligosaccharides were moderate and were found for *LALBA* with 6′-SL (r = − 0.43280; *p* = 0.0033), 3′-SL (r = − 054178; *p* = 0.0001), and DSL (r = − 0.64733; p < 0.0001).

## Discussion

To our knowledge, this study is the first to investigate the gene expression of candidate genes for OS biosynthesis in different goat breeds during four lactation stages. In fact, only one RNA-seq experiment in the Jersey and Friesian bovine breeds [37] and one in the Maltese goat breed [38] have been reported to date. The identification of the candidate gene cDNAs represents a first step toward gene expression analysis.

The mutations found in the *ST3GAL5* coding cDNA lead to amino acid changes in the protein transmembrane domain (TM).

Compared with other Golgi-resident glycosyltransferases (GT), the deduced proteins of both *ST3GAL5* and *ST6GAL1* shared a typical type II membrane protein topology (consisting of a short N-terminal cytoplasmic tail, a transmembrane (TM) domain followed by a stem region and a large C-terminal catalytic domain facing the luminal side) and sialyl motifs in their catalytic domain [39, 40].

The Phe76Ser change (c.240 T > C) identified in the *ST3GAL5* cDNA is located in the middle of the sequence, and SIFT prediction showed the mutation to be deleterious for protein function. The N-terminal region of glycosyltransferases, and particularly the transmembrane domain, is crucial for the correct Golgi localization of the enzymes [41]. a change from an apolar to a polar in amino acid residue the hydrophobic TM could interfere with the correct localization of an enzyme and, consequently, its function. In humans, a missense change in the TM region of an *ST3GAL3* family protein was shown to co-segregate with intellectual disability, and cellular and biochemical systems showed that this mutation caused ER retention and drastically impaired protein functionality [42]. It would be interesting to investigate the c.240 T > C mutation more deeply in at least the two goat breeds examined herein and in a larger number of animals to uncover its potential influence on gene expression.

The Ser69Thr amino acid change (caused by the c.372 T > A SNP) found in the *ST6GAL1* cDNA is located in the stem region outside of the catalytic region of the protein, and SIFT analysis did not show deleterious effects on protein function. Considering the preliminary identification of *ST6GALT1* splicing variants in GMSCs, the obtained bands were less distinct compared with the principal band. Moreover, the higher band gives rise to a deduced protein product lacking the second coding exon containing the entire L sialyl motif of the catalytic domain, while the lower band gives rise to a protein lacking the TM domain. Thus, these bands would not produce a complete protein, although the full transcript could compensate for this, or they might represent expressed pseudogene forms. as reported for bovines [43].

The whey α-lactalbumin protein coding gene (*LALBA*) showed the highest expression in our experiment, which agrees with previous findings in goats [44], cows [45], sheep [46] and humans [47]. The increase in *LALBA* expression that we observed starting from the colostrum stage was consistent with increases in both the milk protein output and lactose synthesis. LALBA is a major protein component of whey, and a high level of *LALBA* expression in mammary epithelial cells is required during lactation. At 120 days, *LALBA* values were shown to be high in MA, while in GA, they tended to decrease; we hypothesize that this trend could be related to the longer lactation period of the MA than the GA breed. In the mammary gland, B4GalT-1 interacts with the α-lactalbumin protein to form the lactose synthase complex which is involved in the final step of lactose synthesis by transferring the galactose moiety of free UDP-Gal to Glc rather than to GlcNAc [7]. Although this process has been demonstrated to occur in intact Golgi, the precise mechanisms whereby lactose is modified by the addition of other sugars by specific glycosyltransferases during lactation are still unknown. It is assumed that the same enzymes involved in producing the termini of other glycan classes might be responsible. The *B4GALT1*-encoded protein is one of the best-studied galactosyltransferase enzymes; it is a trans-Golgi resident type II membrane-bound glycoprotein that catalyses the transfer of galactose to N-acetylglucosamine residues and participates in the biosynthesis of the oligosaccharide structures of glycans. Since B4GalT-1 has been identified on the plasma membrane, it may also be involved in cell-cell or cell-substrate recognition [48]. *B4GALT1* gene expression was maintained at similar levels during lactation in the GA breed, while increasing expression values were shown in the MA breed; the latter is in agreement with the results reported in an RNA-seq experiment in goat [38].

The expression patterns of both *LALBA* and *B4GALT1* suggest that the encoded proteins are present during lactation and are assembled to carry out the production of lactose, which might in turn be used as a precursor for SOS biosynthesis. A moderate positive correlation coefficient between the *B4GALT1* and *LALBA* genes was found in the experiment, supporting our hypothesis.

Considering the *ST6GAL1* gene, its expression in bovines is primarily regulated at the transcription level by several cell and development-specific promoters, producing transcripts with divergent 5′ untranslated regions (UTR) [43]. In the mouse mammary gland [49], a novel mRNA isoform of the *ST6GALI* gene containing a novel 5′UTR exon (L) that is associated with a drastic increase in gene expression during lactation has been identified. Dalziel et al. [50] found a similar transcript in the rat mammary gland. In contrast, no exon (L)-containing transcript was detected in the lactating bovine or human mammary gland. The authors also observed a trend of increasing *ST6GAL1* gene expression in the bovine mammary gland, culminating in involution. This contrasts with findings in species such as mice, in which the greatest change in *ST6GAL1* gene expression occurs between pregnancy and lactation, suggesting different roles in rodents vs. other mammals. The transcripts identified in this study showed differences in their coding regions, and we could not clarify the nature of the different 5′ regions of the transcripts because neither 5’RACE or a promoter analysis was performed.

The mammary gland is susceptible to modification and remodelling associated with cell turnover and apoptosis during lactation [51, 52]. On our experimental farm, the goats are extensively reared, and kids are left with parents for a long period until the peak of lactation, when they are separated. At the beginning of weaning in the mammary gland, some modifications lead to involution; Li et al. [53] found that 5% of cells in the involuting tissue in goats were apoptotic, whereas less than 1% of cells in lactating tissue were undergoing death. During this phase, greater susceptibility to infection arises, with the consequent stimulation of the immune system, which plays an important role in the defence of the mammary gland. The review of Mills et al. [54] explains the possible roles of OS that harbour epitopes similar to selectin-binding ligands and may play a role in anti-inflammatory processes.

We hypothesize that at 60 days, both the *ST6GAL1* and *ST3GAL5* genes might be expressed at higher levels due to their potential involvement in the synthesis of glycoconjugate sialylated structures, which are fundamental for the immune response.

Sialyltransferases are divided into four families, ST6Gal, ST3Gal, ST6GalNAc and ST8Sia, according to the glycosidic linkage formed and the monosaccharide acceptor used. Currently, 20 sialyltransferase sub-families are known in higher vertebrates [23]. The ST3gal gene family comprises six ST3Gal sub-families, and the ST6Gal gene family comprises two sub-families. ST3Gal III, IV, V, and VI use the oligosaccharide isomers Gal β1–3/4Glc(NAc)-R.

RNA-seq studies in both goats [38] and bovines [37] showed the expression of all 6 sub-family ST3Gal genes, including *ST3GAL5* and *ST6GAL1*. In goats, the most highly expressed genes were *ST3GAL1, ST3GAL4* and *ST3GAL6.* Another study [55] found that the most highly expressed genes in mouse mammary glands were *ST3GAL1, ST3GAL4* and *ST6GAL1*. By using knock-out mice for these genes and analysing 3′-SL and 6′SL levels in milk, it was shown that the *ST3GAL4* and *ST6GAL1* genes account for the bulk of 3′SL and 6′-SL production, respectively.

When we looked at the possible correlations of the phenotypes analysed in the experiment, we found positive correlations among genes and among SOS. A positive correlation of *ST3GAL5* and *ST6GAL1* expression with 3′-SL and 6′-SL colostrum/milk contents was not found. In fact, the concentration of SOS was initially high in colostrum (0 and 1 day) and then decreased, in accordance with the literature [11, 32, 56, 57], while for gene expression, the opposite pattern was observed.

The lack of association between some of the chosen candidate genes and SOS content might have occurred because other sialyltransferase genes such as *ST3GAL4* or *ST3GAL6* [58] might be involved in the biosynthesis of the free OS fraction.

Overall, the higher expression of *ST3GALT5* to *ST6GALT1* found in this study corresponds to the higher concentration of 3′-SL compared to 6′-SL, which is supported by Crisà et al. [38] and Wickramasinghe et al. [37], who reported the same differences in gene expression in RNA-seq experiments in goats and cows.

The differences in SOS contents between the GA and MA goat breeds have been investigated by Claps et al. [32], although they sampled milk at different time points than were examined in the present study, and we could perform comparisons only for the results for 0 and 1 days. In their experiment, DSL showed the third highest mean value among the SOS after 3′-SL and 6′SL, and GA colostrum and milk contained higher levels of 3′-SL, 6′-SL and DSL than those of MA. In our experiment, DSL was the second most concentrated SOS after 3′SL, and the proportions of SOS between GA and MA differed on the different days. Only on day 1 did 3′-SL present a significantly higher value in GA, while DSL presented a higher value in MA. Given that DSL is formed from 3′-SL, we speculate that in MA, a fraction of 3′-SL might be used to synthesize DSL. This suggestion was corroborated by the results of correlation analyses in which higher and more statistically positive coefficients were found between 3′-SL and DSL than between 6′-SL and DSL.

Furthermore, we do not have data on the expression of *ST8SIA* genes that could support this suggestion. In mice, it was shown that the ST8SiaVI product exhibited higher activity towards 3′-SL than towards 6′-SL [26].

A limitation of our study is that we considered only certain putative genes involved in the last step of SOS biosynthesis while not taking into account precursor availability for the sialic acid and lactose synthesis pathways. The goats were fed the same diet, so the diet would not be an issue explaining the observed differences, which could instead reflect an effect of parity (differences between experimental animals), season (from February to June), the duration of lactation (which is shorter in the GA breed) or production output (which is lower in the GA breed) effects. Moreover, we are aware that other intermediate biological processes exist between gene expression and the production of functional proteins.

## Conclusions

This study represents the first attempt in investigating the mRNA expression profiles of candidate genes implicated in SOS biosynthesis in different goat breeds during lactation. Genetic effects on the OS content of milk have been observed in bovines, and we report candidate gene expression variability in two breeds of goats, one of which is autochthonous and economically important to the Gargano promontory. Moreover, the higher expression of *ST3GALT5* with respect to *ST6GALT1* corresponds to what has been reported in the literature regarding the higher concentration of 3′-SL compared to 6′-SL in goat colostrum and milk. However, no clear correlation between genes and SOS contents in colostrum and milk was shown by the analysis. Although the investigation of additional sialyltransferase genes that support and explain SOS production is needed, our findings highlight the presence of SNPs in the studied genes, one of which might impair the protein cellular localization and function of ST3GAL5. The identification of different *ST6GAL1* transcripts suggests the existence of a complex regulatory process in the promoter region, as observed in other mammalian species. The investigation of SNPs in the wider Maltese and Garganica population as well the gene regulatory regions, which may account for different transcripts or expression levels, will be needed to identify associations with OS milk content. Garganica is an important autochthonous goat breed, especially for interior and mountainous areas, and the identification of genetic patterns leading to higher SOS contents might be useful for breeding purposes and a key element for breed protection.

## Methods

### Animals and sample collection

This study was conducted on the experimental farm of CREA (Consiglio per la Ricerca in Agricoltura e l’Analisi dell’Economia Agraria, Research Centre for Animal Production and Acquaculture, Bella (PZ)), a research institute authorized by the Italian Ministry of Health to use farm animals for experimental purposes (DM 26/96–4). Animal management and care followed the recommendations of European Union directive 86/609/EEC.

A dairy breed, Maltese (MA) goats, and a less productive breed, Garganica goats (GA), were considered in this trial, including a total of 18 does (9 GA and 9 MA). The animals were fed indoors, receiving mixed meadow hay ad libitum plus concentrate supplementation at 14% of crude protein (CP), which was 600 g/d for MA and 400 g/d for GA in relation to their level of milk production. Within each breed, the animals were grouped homogenously according to their live weight (46.1 kg and 48.6 kg on average for MA and GA, respectively) and milk production (1200 and 800 g/day of milk, respectively, in mid lactation).

At four lactation stages (colostrum (after kidding and day 1), 60 and 120 days), individual colostrum/milk samples were collected in a 50 ml tube and transported to the laboratory at 4 °C for RNA extraction. For OS determination (3′-SL, 6′-SL, DSL), the samples were distributed in 10 ml tubes and immediately frozen at − 20 °C until analysis. At the fourth lactation stage, some samples were not collected because the ewes had run dry.

### RNA and DNA extraction

RNA was obtained from goat milk somatic cells (GMSCs) in accord with both [59], who showed that GMSCs can be used to accurately reveal the cellular dynamics of mammary gland gene expression, and other authors [60, 61] who demonstrated extensive similarity between the mammary gland and MSC/ mammary epithelial cell transcriptomes. RNA was extracted from somatic cells obtained from 50 ml of goat colostrum or milk in a total of 72 samples. Individual samples were immediately centrifuged at 2000 g for 10 min at 4 °C in the presence of 50 μl EDTA 0.5 M pH 8. The fat layer on the top of the supernatant was removed with a sterile pipette tip, the skim milk was discarded, and the somatic cell pellet was resuspended in 1 ml of 1X PBS with 300 μl of 0.5 M pH 8 EDTA and centrifuged again. The resulting cells were lysed with 1 ml TriReagent (Sigma-Aldrich, Milan, Italy). Total RNA extraction was performed following the manufacturer’s instructions. RNA was digested to remove contaminated DNAs with an RNase-Free DNase Set (Qiagen) and successfully cleaned with an RNeasy MinElute kit (Qiagen, Milan, Italy). RNA quality and quantity were checked via a spectrophotometry (NanoPhotometer™ Pearl, Implen GmbH, München, Germany) and microfluidic (2100 Bioanalyzer, Agilent Technologies, Milan, Italy) inspection to assess RNA integrity. The RNA samples were stored at − 80 °C. Only RNA with an RNA Integrity Number (RIN) > 7 was used in the gene expression analyses (6 animals of each breed, total of 45 samples). DNA was extracted from the interphase obtained from milk cell pellets following the TriReagent (Sigma-Aldrich, Milan, Italy) manufacturer’s instructions.

### Reverse transcription into cDNA and sequencing

RNA was reverse transcribed to produce both long cDNAs and cDNAs for qPCR. In the first case, the Maxima H Minus First Strand cDNA Synthesis kit (Thermo Scientific, Thermo Fisher Scientific, Waltham, USA) was employed starting from 1 μg of RNA in a total volume of 20 μl containing 100 pmol oligo (dT) (18-mer), 0.5 mM dNTPs, 5X RT buffer, and Maxima H Minus Enzyme mix according to the manufacturer’s instructions. The resulting cDNA was diluted 1:2 and PCR amplified by using the primer pairs listed in Table 1. PCR amplification was performed using Dream Taq DNA polymerase (Thermo Scientific, Thermo Fisher Scientific, Waltham, USA) with 1 μl of the first-strand cDNA reaction and a touchdown protocol. The PCR products were gel purified using Nucleospin columns (Macherey-Nagel, GmbH & Co KG, Duren, Germany) and bi-directionally sequenced by using the BidDye Terminator v. 1.1 Cycle Sequencing kit and an ABI 3700 sequencer (Applied Biosystems, Life Technologies, Milan, Italy).

For the cDNAs to be used in gene expression experiments, the Maxima First Strand cDNA synthesis kit for RT-qPCR (Thermo Scientific, Thermo Fisher Scientific, Waltham, USA) was used. Briefly, RT reactions were prepared by adding 500 ng RNA, 4 μl of a 5X reaction mix (containing random hexamers and oligo (dT)_18,_ and 2 μl of enzyme mix in a final volume of 20 μl. The reaction was incubated at 25 °C for 10 min, then at 50 °C for 15 min, and finally at 85 °C for 5 min. An RT-negative reaction was set up to check for genomic DNA contamination. The cDNA was first diluted 1:4 and then used directly for qPCR or stored at − 20 °C.

### Primer design and sequence analysis

At the beginning of our study, no annotations for the studied genes in goats were present in GenBank except for *LALBA* (NM_001285635). Primers were picked by using Primer 3 software [62, 63] on the basis of known bovine sequences. After initial trials with different primer pairs, the complete coding sequences of *ST6GAL1*, *ST3GAL5*, and *B4GALT1* were obtained by merging the sequences from two or three amplicons (Table 6). Sequence analysis and alignments (nucleotidic and proteic deduced ones) were performed using DNAMAN (Version 4.15, Lynnon BioSoft, Inc., San Ramon, USA), Bio Edit software [64] and the BLAST tool [65, 66].

**Table 6:** Primer sequences, reference sequences, position in the reference, T.a. and amplicon size of target genes

Gene symbol	Primer sequences	Reference sequence	Position	T.a.	Amplicon *base pair*
*B4GALT1*	FW-5’TAAAGCGGCGGCGGGAAGAT3’ RW-‘5AATGAGAGGGACCAGCCCA3’	NM_177512.2	166–185 1460–1478	57	1313
*B4GALT1*	FW-5’GTTTCTCAGCATCAATGGATTTCC3’ RW-5’GCTTTGATTCTTTGGGGTGA3’	NM_177512.2	1083–1106 1652–1672	58	590
*ST3GAL5*	FW-5’CCCGCTCCCTAATATGCGAA3’ RW-5’TGCGATCAGGATCCATGTAA3’	NM_205807	30–49 342–361	58	332
*ST3GAL5*	FW-5’AATGCCAAGTGACCACAGC3’ RW-5’CGACATCAAACTGGTTCAGG3’	NM_205807	129–147 684–704	58	460
*ST3GAL5*	FW-5’CCTGAACCAGTTTGATGTC3’ RW-5’CAGCATTGGAAGCACAGAGT3’	NM_205807	684–704 1369–1389	58	705
*ST6GAL1*	FW-5’TCCTGAGAAGAATGAGCCTTG3’ RW-5’AGTGAGACAGGAGGCTCTGG3’	NM_177517	15–35 1597–1617	56	1593
*ST6GAL1*	FW-5’TGAAGTACCTCAACCTCGGCA3’ RW-5’GGGAAAGGTGGATCTTGCG3’	NM_177517	1307–1329 1677–1696	58	379

T.a. = annealing temperature

SIFT software analysis was performed to predict whether an amino acid substitution affects protein function based on sequence homology and the physical properties of amino acids [67].

Primer Express software (Applied Biosystem, Thermo Fisher Scientific, Waltham, USA) was used to design primers for qPCR on the basis of bovine sequences. Five reference genes (RGs) selected in another study [68] were tested here: ATP synthase β polypeptide, a nuclear gene encoding a mitochondrial protein (*ATP5B*), eukaryotic translation initiation factor eIF-2B subunit β (*EIF2B2*), succinate dehydrogenase complex subunit A flavoprotein (*SDHA*), DNA-directed RNA polymerase fragment RNA polymerase II (*POLR2A*), and a TATA box-binding protein (*TBP*). We designed and tested new primers for the following RGs: a ubiquitously expressed transcript (*UXT*) and ribosomal protein S9 (*RPS9*). The primers were intron spanning when possible and were synthesized by Eurofins MWG Operon (Ebersberg, Germany). Table 7 provides information related to the primers used to amplify the targets and RGs. Preliminary amplifications were performed on both cDNA and DNA to validate the primers. PCR products were verified via an agarose gel run and sequenced using a 3500 Genetic Analyzer (Applied Biosystem, Thermo Fisher Scientific, Waltham, USA) Amplification specificity was verified by using the BLAST tool [65].

**Table 7:** Primer sequences, reference sequences, covered regions and positions in the mRNAs, and expected amplicon sizes for both the target and reference genes used in the qPCR analysis

Gene symbol	Primer sequences	Reference sequence	Covered region / Position	Amplicon, *base pairs*
*B4GALT1*	FW-5’CTGTGTCTCGCCCAAATGCT3’ RW-5’AGGTGAGTGAGTTCAAACCATCAG3’	HQ700335.1	Ex5fw/1011–1030 Ex6rw/1134–1157	147
*LALBA*	FW-5’GATGACATTGTGTGTGCCAAGA3’ RW-5’AGTGCCACTGATCCAGCTTCTC3’	NM_001285635.1	Ex3fw/330–351 Ex4rw/408–429	100
*ST3GAL5*	FW-5’CCTGAACCAGTTTGATGTCG3’ RW-5’GGTGCACCTTCTGGATAAGTC3’	KF055858.2	E4fw/655–673 E5rw/745–765	111
*ST6GAL1*	FW-5’GAGCTGTGGGACATCATTCAAG3’ RW-5’CACACAGTGACATCATAATGGCAAT3’	HQ709167.1	E5fw/1065–1086 E6rw/1140–1164	100
*ATP5B*	FW-5’TTTGGACTCCACGTCTCGCATC3’ RW-5’TCCTGGAGGGATTTGTAGTCCTG3’	NM_175796.2	Ex8fw/1219–1240 Ex9rw/1304–1326	108
*EIF2B2*	FW-5’CCGTTCCCATTATGCTCAACTCCAG3’ RW-5’TCCGTTGTCCCTTCCAGTTCCAC3’	NM_001015593.1	Ex3fw/395–419 Ex3-4rw/453–475	81
*SDHA*	FW-5’ACGATTACTCCAAGCCCATCCAG3’ RW-5’AACGTAGGAGAGCGTGTGCTTC3’	NM_174178.2	Ex14fw/1825–1847 Ex14rw/1883–1904	80
*POLR2A*	FW-5′ AATGGAAGCATGTCAATGAGGACTCTC3’ RW-5′ CACAGGCAGCACAGTGACGATC3’	NM_001206313.1	Ex5fw/602–628 Ex5rw/744–765	164
*TBP*	FW-5’AACAGGTGCTAAAGTCAGAGCAG3’ RW-5′ GGAGAACACAGCAGCCATTACG3’	NM_001075742.1	Ex8fw/1254–1276 Ex9rw/1334–1355	102
*UXT*	FW-5’TGTGGCCCTTGGATATGGTT3’ RW-5’GGTTGTCGCTGAGCTCTGTG3’	NM_001037471.2	Ex5fw/300–319 Ex5-6rw/381–400	101
*RPS9*	FW-5’CCTCGACCAAGAGCTGAAG3’ RW-5’CCTCCAGACCTCACGTTTGTTC3’	NM_001101152.1	Ex2fw/128–146 Ex3rw/170–191	54

### QPCR assay

In gene expression experiments, the suitability of genes to be used as references (RGs) is not given a priori and has to be evaluated each [69]. Preliminary qPCR experiments were performed to optimize the annealing temperature and primer concentration (100–300 nM) of both the target genes and the seven RGs.

The efficiency of PCR amplification for each gene was calculated with a five-point standard curve (1:5 dilution per point) generated on the basis of cDNA samples used as a calibrator. QPCR was conducted using Max SYBR Green/ROX qPCR Master Mix (Thermo Scientific, Thermo Fisher Scientific, Waltham, USA) in a StepOne Plus instrument (Applied Biosystems, Thermo Fisher Scientific, Waltham, USA). Each reaction was run in triplicate and contained 10 ng of cDNA template along with the specific corresponding primer concentrations and 10 μl of 2X master mix in a final reaction volume of 20 μl. Reverse transcriptase minus (RT-) and no template controls (NTC) were included. The cycling parameters were 95° for 10 min to activate the DNA polymerase, followed by 40 cycles of 95° for 30 s and T.a. for 1 min. To verify that the primer pairs produced only a single product, a dissociation protocol from 65 °C to 95 °C was added after thermocycling.

### Oligosaccharide isolation and HPAEC analysis

OS were isolated from individual colostrum and milk samples as described by Mc Jarrow and Van Amelsfort-Schoonbeek [70]. Briefly, after centrifugation at 2000×g at 4 °C for 10 min, the supernatant lipid layer was removed, and the proteins were precipitated by the addition of 0.5 volumes of 1.8 g 100 mL^− 1^ Ba (OH)_2_·8H_2_O and 0.5 volumes of 2 g 100 mL^− 1^ ZnSO_4_·7H_2_O. The blend was mixed by vortexing and centrifuged at 12.000×g in a microfuge for 10 min at 4 °C. The supernatant was carefully removed and centrifuged again. The second supernatant was filtered with a nylon filter with a 0.45 μm pore size. The total OS fraction was separated using high-performance anion-exchange chromatography (HPAEC) in a Dionex PA100 column (Dionex, Sunnyvale, California, USA). The eluted fractions were monitored by pulsed amperometric detection (Dionex ED40), and the gradient was controlled by a Varian Pro Star pump system, which was capable of maintaining a flow rate of 1 ml/min for the duration of the run. Data were collected and analysed with Star Chromatography Workstation 6.41 (Varian, Inc. Walnut Creek, California, USA), and 6′-SL, 3′-SL, and DSL external standards were used to generate standard curves for comparison.

### QPCR data analysis and statistics

The results obtained with Sequence Detection Software (Applied Biosystems, version 2.1, Thermo Fisher Scientific, Waltham, USA)) were exported as tab-delimited text files and imported into qBase^PLUS^ software. qBase^PLUS^ included a geNorm^PLUS^ analysis to screen the optimal set of reference candidate genes [71]. The geNormplus analysis was assessed by using the seven tested candidates and nine samples belonging to four lactation stages. geNorm calculates the gene expression stability measure (M) for a reference gene as the average “pairwise variation” (V) for that gene in relation to all other tested reference genes. Stepwise exclusion of the gene with the highest M value allows the ranking of the tested genes according to their expression stability.

The qBase^PLUS^ software (version 2.5. Biogazelle, Gent, Belgium) was used to the analyse qPCR data based on the 2^-∆∆Ct^ method, with implementations to take multiple reference genes and gene-specific amplification efficiencies into account as well as the errors of all measured parameters along the entire calculation track. Moreover, an inter-run calibration algorithm is included to correct for run-to-run differences [72]. Cumulative normalized relative quantities (CNRQs) represent n-fold normalized expression relative to inter-run calibrators (IRC) run across all plates. The logarithm (Log) of the CNRQs was used for graphical representation and statistical analysis.

Least square (LS) means for the breeds, lactation stages and oligosaccharide contents were calculated with Proc GLM in SAS software (SAS Institute Inc., 2010) using the following model:

y_ijk_ = *μ* + C_j_ + K_i_ + (C_j_*K_i_) + e_ijk_where:

y_ijk_ = phenotype (gene expression or OS content) of the i^th^ goat at the k^th^ lactation stage in the j^th^ breed;

C_j_ = fixed effect of the j^th^ breed (j = 1–2);

K_i_ = fixed effect of the i^th^ stage of lactation (i = 1–4);

e_ijk_ = random residual effect.

Pearson correlation coefficients (r) for breed, lactation stages and oligosaccharide content were calculated using Proc CORR in SAS software (SAS Institute Inc., 2010).

The significance of the analyses was determined using Tukey’s test under the GLM procedure.

## Supplementary information

**Additional file 1:** Primer concentration, annealing temperatures and standard curve qPCR parameters. Parameters used for the setup of the qPCR experiment for the reference and target genes.

**Additional file 2:** Results of geNormPlus analysis. The figure represents the most stable reference genes suggested by geNorm for qPCR experiments starting from the right side and moving to the left.

## Data Availability

Data generated during this study are included in this published article [and its supplementary information files]. cDNA sequences generated during the current study are available in the GenBank repository [https://www.ncbi.nlm.nih.gov/genbank/] under accession numbers: HQ700335.1, KF055858.2, HQ709167.1.

## Abbreviations

3′-SL: 3’sialyllactose
6′-SL: 6’sialyllactose
GMSCs: Goat milk somatic cells
OS: Oligosaccharides
SOS: Sialyloligosaccharides
