# Supplementary material for: Identification of the complete coding cDNAs and expression analysis of B4GALT1, LALBA, ST3GAL5, ST6GAL1 in the colostrum and milk of the Garganica and Maltese goat breeds to reveal possible implications for oligosaccharide biosynthesis
Source: BMC Vet Res. 2019 Dec 18;15:457. doi: 10.1186/s12917-019-2206-0 (PMC6921551; doi:10.1186/s12917-019-2206-0)
Supplement: Supplementary file 1 — Additional file 1: Primer concentration, annealing temperatures and standard curve qPCR parameters. Parameters used for the setup of the qPCR experiment for the reference and target genes. [file 12917_2019_2206_MOESM1_ESM.docx]

**Additional file 1. Primer concentration, annealing temperatures (T.a.) and standard curves qPCR parameters**.

| **Gene name** | **Primer,** nM | **T.a.** | **Slope ^1^** | **Y-intercept** | **(R^2^)^2^** | **Efficiency, % ^3^** |
| --- | --- | --- | --- | --- | --- | --- |
| *B4GALT1* | 200/200 | 61 | -3,410 | 26,38 | 0,99 | 96,4 |
| *LALB* | 300/300 | 60 | -3,52 | 21,59 | 1 | 92,5 |
| *ST3GAL5* | 300/300 | 60 | -3,20 | 29,59 | 0,99 | 105 |
| *ST6GAL1* | 100/300 | 61 | -3,52 | 28,28 | 0,99 | 92 |
| *ATP5B* | 300/300 | 61 | -3,37 | 25,61 | 0,99 | 98 |
| *EIF2B2* | 200/200 | 61 | -3,61 | 30,22 | 0,99 | 89 |
| *POLR2A* | 100/100 | 61 | -3,62 | 25,98 | 0,99 | 90 |
| *SDHA* | 200/100 | 61 | -3,31 | 27,25 | 0,99 | 97 |
| *TBP* | 100/300 | 58 | -3,26 | 31,60 | 0,97 | 102 |
| *UXT* | 300/300 | 58 | -3,29 | 29,29 | 0,99 | 101 |
| *RPS9* | 200/200 | 58 | -3,33 | 24,57 | 1 | 99,5 |

1 Slope of the standard curve

2 R2= coefficient of determination of the standard curve

3 Calculated as [10(-1/Slope)]
