# Supplementary figures and images for: Identification of the complete coding cDNAs and expression analysis of B4GALT1, LALBA, ST3GAL5, ST6GAL1 in the colostrum and milk of the Garganica and Maltese goat breeds to reveal possible implications for oligosaccharide biosynthesis
Source: BMC Vet Res. 2019 Dec 18;15:457. doi: 10.1186/s12917-019-2206-0 (PMC6921551; doi:10.1186/s12917-019-2206-0)

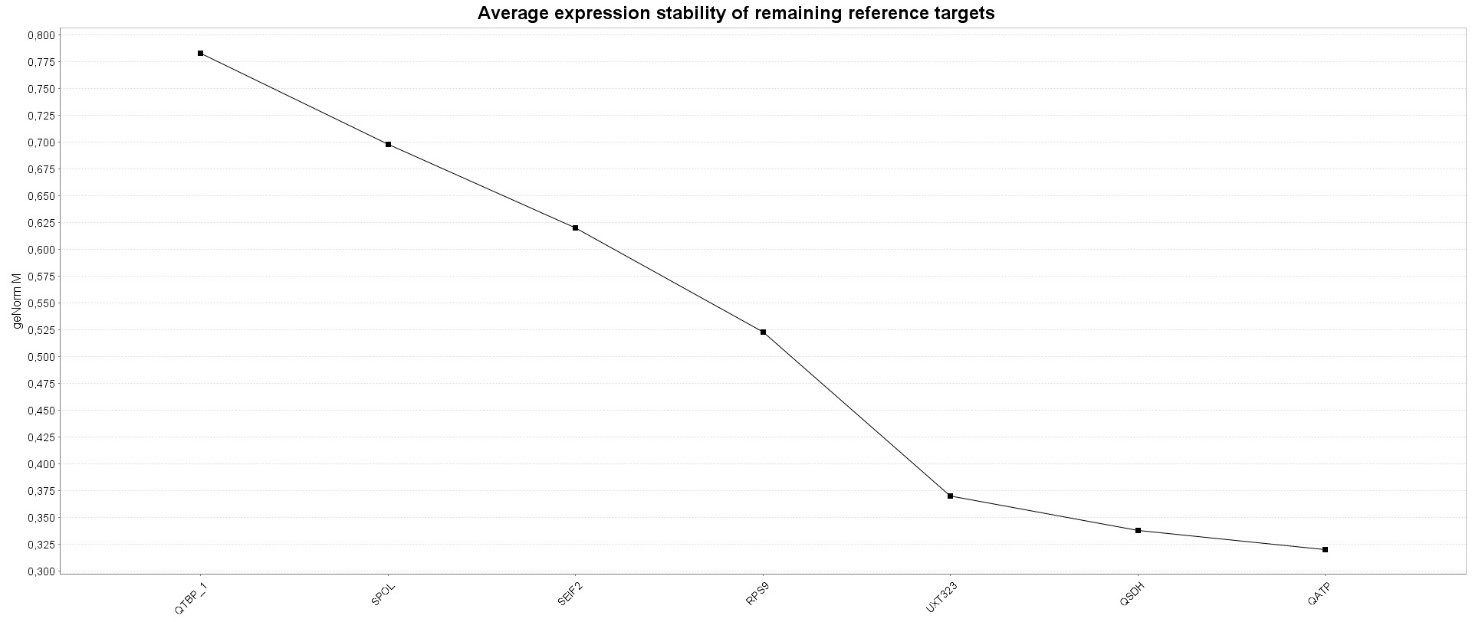

Supplement: Supplementary file 2 — Additional file 2: Results of geNormPlus analysis. The figure represents the most stable reference genes suggested by geNorm for qPCR experiments starting from the right side and moving to the left. [file 12917_2019_2206_MOESM2_ESM.jpg]
